# Improvement of the Force Field for *β*-d-Glucose with Machine Learning

**DOI:** 10.3390/molecules26216691

**Published:** 2021-11-05

**Authors:** Makoto Ikejo, Hirofumi Watanabe, Kohei Shimamura, Shigenori Tanaka

**Affiliations:** 1 Department of Computational Science, Graduate School of System Informatics, Kobe University, 1-1 Rokkodai-cho, Nada-ku, Kobe 657-8501, Japan; shimamura@kumamoto-u.ac.jp; 2 WithMetis Co., Ltd., Wembley Building 7th Floor, 6-1-17 Isogami-dori, Chuo-ku, Kobe 651-0086, Japan; h.watanabe314@gmail.com; 3 Department of Physics, Kumamoto University, Kumamoto 860-8555, Japan

**Keywords:** force field, glucose, machine learning, molecular dynamics, GLYCAM

## Abstract

While the construction of a dependable force field for performing classical molecular dynamics (MD) simulation is crucial for elucidating the structure and function of biomolecular systems, the attempts to do this for glycans are relatively sparse compared to those for proteins and nucleic acids. Currently, the use of GLYCAM06 force field is the most popular, but there have been a number of concerns about its accuracy in the systematic description of structural changes. In the present work, we focus on the improvement of the GLYCAM06 force field for β-d-glucose, a simple and the most abundant monosaccharide molecule, with the aid of machine learning techniques implemented with the TensorFlow library. Following the pre-sampling over a wide range of configuration space generated by MD simulation, the atomic charge and dihedral angle parameters in the GLYCAM06 force field were re-optimized to accurately reproduce the relative energies of β-d-glucose obtained by the density functional theory (DFT) calculations according to the structural changes. The validation for the newly proposed force-field parameters was then carried out by verifying that the relative energy errors compared to the DFT value were significantly reduced and that some inconsistencies with experimental (e.g., NMR) results observed in the GLYCAM06 force field were resolved relevantly.

## 1. Introduction

Glycans, the third commonest group of biomolecules after proteins and nucleic acids, are involved in cell differentiation [1], cell-to-cell recognition [2], and colonization and entry processes of viruses and bacteria into host cells [3]. It is scientifically important to elucidate the function of glycans; therefore, it is essential to clarify their structure and dynamics. X-ray-based crystal structure analysis has been widely used for structural analysis of proteins. However, as for the glycans attached to proteins, X-ray-based crystal structure analysis shows that (i) glycans are often removed by enzymatic deglycosylation treatment to obtain a single crystal, and (ii) it is difficult to identify their electron density, owing to their heterogeneity and conformational diversity [4]. For these reasons, information about the three-dimensional structure of glycans is sparse, compared with that for proteins. Therefore, computer-based analysis of the structure and function of glycans is important. In particular, molecular dynamics (MD) simulation-based analysis has generated a significant amount of knowledge regarding glycans [5,6,7,8]. However, the performance of the MD method strongly depends on the force field determination accuracy [9,10,11]; hence, accurate force field determination has been important in glycan research.

Methods for protein force field determination are widely established; various approaches, such as charge determination methods [12,13,14] and dihedral angle treatment [15] have been proposed. For adjusting the side-chain dihedral angle parameter of the ff14SB force field [16] used in the MD package AMBER [17,18], parametric fitting was performed so that the force field calculation reproduced the energy changes in the quantum mechanical (QM) calculations owing to structural changes. In addition, as a method for verifying the relevance of parameters, a comparison of the respective ${}^{3}J$ coupling constants obtained from nuclear magnetic resonance (NMR) experiments and MD calculations was performed [16]. Furthermore, in recent years, machine learning methods have also been used for parameter fitting [19,20,21].

On the other hand, in the GLYCAM06 force field framework [22] developed as a force field for glycans in the AMBER package, the force field parameters were optimized for reproducing the QM energy with B3LYP/6-31++G(2d,2p) accuracy, using small organic molecules as model systems. However, according to the report by Marianski et al. [23], when the relative energy of glucose was compared between the highly accurate QM calculations and the molecular mechanics (MM) calculations using the GLYCAM06 force field, the energy accuracy was found to be fairly poor. In addition, comparing the NMR measurement results by Nishida et al. [24] with the MD simulation results for 50 ns (described below), the distributions of the occurrence frequencies of $C_{5}$–$C_{6}$ bond rotamers showed different tendencies: Regarding the orientation of the 6-position hydroxymethyl group, a tendency has been found that the gg conformation had a larger or similar proportion than the gt conformation in experiment, whereas the gt proportion was larger in the MD calculation using the GLYCAM06 force field.

In order to overcome these problems associated with constructing a reliable force field for glycans, β-glucose, which is the simplest monosaccharide structure, was employed as a target molecule in the first step. In this study, one β-glucose molecule was used as a model molecule for parameter fitting instead of small organic molecules as in the GLYCAM06 force field. We would like to replace the parameters in the GLYCAM06 force field with improved ones step by step, and at least for β-glucose, we have found an improvement over GLYCAM06 from the viewpoint of energy evaluation. Our final goal in improving the force field is to create more accurate force-field parameters for each monosaccharide and to re-optimize the dihedral-angle parameters for the glycosidic linkage based on these parameters, thus leading to a total improvement on the glycan force field. For the β-d-glucose, structural sampling was performed using the MD method, and the dihedral angle and atomic charge force-field parameters were fitted for minimizing the energy error between the QM and force field calculations. Furthermore, to validate the force field estimation, MD simulations were performed using the GLYCAM06 force field and that developed in the current research, and the results were compared with the NMR experimental values and the energies obtained by QM calculations. In this study, we employed a machine learning method to fit multiple dihedral angles and atomic charge parameters, thus opening a new avenue for the development of glycan force field. The employed approach is described in details below.

## 2. Materials and Methods

**Figure 1:**
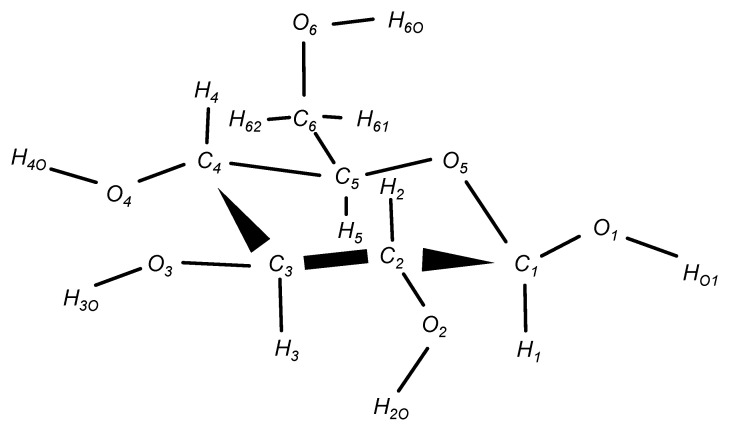
Molecular structure of β-glucose. The atomic labels are the same as those for the GLYCAM06 force field [22], and correspond to the atomic labels in the text.

In this study, we targeted the monosaccharide β-glucose molecule, which has the most typical and simple structure. The chemical structure of the β-glucose molecule is shown in Figure 1, in which the labels of atoms are defined. AMBER14 [17] and AmberTools16 [18] were used for MD simulations and force field calculations, respectively, and Gaussian09 [25] and Gaussian16 [26] were used for QM calculations.

Below, the process of the force field reoptimization and verification in this study is briefly explained. First, the MD simulations were performed using the conventional GLYCAM06 force field [22] for sampling the structure of the β-glucose molecule used for parametric fitting. Next, the energy values for the QM and MM calculations for each structure were obtained, and the relative energy errors with respect to the reference structure were evaluated. All the QM calculated energies in this work are the electronic energies in vacuum. The “relative energies” used in the following calculations are the relative differences in energy from that of the reference structure caused by the deformation of the cyclic β-d-glucose ring structure and the change in the orientation of the exocyclic functional groups. The reference structures are defined in Section 2.1 and Section 2.8. Furthermore, the force field parameters subject to the optimization were defined for the dihedral angles and atomic charges, and parametric fitting was performed with the objective of minimizing the root mean square of the relative energy error. The existing GLYCAM06 force field values were used for bond-stretching, angle-bending, and Lennard-Jones potential parameters except for the dihedral angle and atomic charge parameters. Finally, the optimized force field parameters were validated by comparing the results obtained using the MM and QM calculations. All of the steps are described in detail below.

### 2.1. Structure Sampling by the GLYCAM06 Force Fields

First, MD simulations using the existing GLYCAM06 force field [22] were performed for structural sampling to optimize the force field parameters. For these MD simulations, we first considered a system consisting of one β-glucose molecule as the solute, and 1716 molecules of water as the solvent, using the tLEaP program of Amber Tools. Then, we minimized the energy of this system for structural optimization. The minimization process consisted of 1500 iterations of the steepest descent algorithm, followed by the application of the conjugate gradient method with at most 50,000 iterations. Subsequently, the system’s temperature was raised to 300 K, and a 50-ns-long production run was performed under the NVT conditions after 2-ns-long temperature relaxation; a total of 1000 structures were sampled every 50 ps. During the MD simulation, covalent bonds containing hydrogen atoms were constrained by the SHAKE algorithm. In addition, temperature was controlled using the Langevin thermostat, and the cutoff distance of the non-bonding interactions was 4.0 Å in this case of structural sampling (see also Section 2.8 below). The particle mesh Ewald (PME) method [27] was used for computing long-range electrostatic interactions. At 300 K, only the most stable six-membered ring structure, called the ${}^{4}C_{1}$ chair structure, was obtained. To obtain metastable structures other than the ${}^{4}C_{1}$ chair structure as well, MD simulations were also performed at 600 K, and structural sampling was performed using the same method as above. The force fields used in the both MD simulations were GLYCAM06 [22] for the β-glucose molecule and TIP3P [28] for water. The structural sampling by MD in this study was intended to obtain the initial structures for the structural optimization by QM, and the cutoff distance for long-range interactions was set to be as small as 4.0 Å to reduce the computational cost. Since the bias of the initial structure was eliminated through the following structural optimization by QM, it was not expected to have a significant impact on the validity of this study.

### 2.2. Structural Optimization Using the QM Method

Next, to eliminate the structural bias owing to the classical force field, the structures were optimized using QM calculations. Water molecules were removed from the snapshots sampled in the MD simulation, and only the glucose molecule was extracted as the initial structure. With this initial structure, the atoms of the six-membered ring ($C_{1},C_{2},C_{3},C_{4},C_{5},\;\mathrm{and}\;O_{5}$ in Figure 1) were fixed, and the structural optimization was performed in vacuum using the B3LYP/6-31++G(2d,2p) method of the density functional theory (DFT). Those structures obtained in the intermediate steps of the structural optimization by QM were also included as sample structures. As a result, 69,501 structures including the intermediate structures were obtained from 2000 initial structures sampled by MD at 300 K and 600 K.

### 2.3. Removal of Duplicate and Outlier Structures

The cost function for the parameter optimization (to be described later) was based on the root mean square of the relative energy error. When the sample structure includes many specific structures, overfitting is likely to occur, where the relative energy error of each specific structure is successfully minimized, but the overall robustness of the method is negatively affected by the lack of generalizability. To avoid overfitting, similar structures were deleted from the 69,501 considered structures, including intermediate structures that were obtained in the course of the structural optimization process, as described above. For discriminating similar structures, the root mean square deviation (RMSD) was computed for every pair of structures according to Equation (1), where structures with RMSD≤0.05 Å were regarded as similar, and were deduplicated:

(1) $$\begin{array}{c} \mathrm{RMSD}=\sqrt{\frac{\sum_{i=1}^{N}{\left(X_{i}-Y_{i}\right)}^{2}}{N}}. \end{array}$$

Here, *N* is the number of atoms, subscript *i* runs over the structure’s atoms, and $X_{i},Y_{i}$ are the three-dimensional coordinates of the two structures for the *i* atom.

Furthermore, we compared the QM and MM energies using the method described in Section 2.4 below, and found that the structures with QM and MM energies differ significantly compared to the other sample groups. We analyzed the MM energy to specify which term in the MM energy (see Equation (3) below) was causing the error, and confirmed that the error was due to the large Lennard-Jones interaction. We thus considered it inappropriate to include these structures in the training data and excluded them, because the Lennard-Jones potential parameter was not fitted in the present study; that is, it is inappropriate to correct the error derived from the Lennard-Jones potential in terms of other fitting parameters. Finally, a dataset consisting of 31,899 structures was considered for parametric optimization.

### 2.4. Calculation of the Relative Energy Error

The most stable structure, according to the QM calculations, was used as the reference structure, and the relative energy error (REE), for each structure, was defined by Equation (2):

(2) $$\begin{array}{cc} \mathrm{REE}(s) & =\left(E_{\mathrm{QM},s}-E_{\mathrm{QM},\mathrm{ref}}\right)-\left(E_{\mathrm{MM},s}-E_{\mathrm{MM},\mathrm{ref}}\right). \end{array}$$

Here, QM and MM represent the quantum and molecular mechanical calculations, respectively, *s* runs over the sample structures, and ref denotes the reference structure. The QM calculations were performed at the B3LYP/6-31++G(2d, 2p) level used for the structural optimization. On the other hand, the functional form of energy in the MM calculations was a general AMBER function:

(3) $$\begin{array}{cc} E_{\mathrm{total}} & =\sum_{\mathrm{bonds}}k_{b}{\left(r-r_{0}\right)}^{2}\\ \; & +\sum_{\mathrm{angles}}k_{\theta }{\left(\theta -\theta_{0}\right)}^{2}\\ \; & +\sum_{\mathrm{dihedrals}}\sum_{n}V_{\phi ,n}\left[1+\cos\left(n\phi -\gamma_{\phi ,n}\right)\right]\\ \; & +\sum_{1-4\mathrm{pair}}\left[\frac{1}{\mathrm{scnb}}\left(\frac{A_{ij}}{R_{ij}^{12}}-\frac{B_{ij}}{R_{ij}^{6}}\right)+\frac{1}{\mathrm{scee}}\frac{q_{i}q_{j}}{\epsilon R_{ij}}\right]\\ \; & +\sum_{i=1}^{N-1}\sum_{j=i+1}^{N}\left[\frac{A_{ij}}{R_{ij}^{12}}-\frac{B_{ij}}{R_{ij}^{6}}+\frac{q_{i}q_{j}}{\epsilon R_{ij}}\right]. \end{array}$$

In the equation above, the first term corresponds to the bond-stretching energy, the second term is the angle-bending energy, the third term is the torsion energy, and the fourth term is the 1–4 interaction. On the other hand, the fifth term refers to the non-bonded interaction, which includes the van der Waals (vdW) interaction and the Coulomb interaction. In this expression, $k_{b},\;r_{0},\;k_{\theta },\;\theta_{0},\;V_{\phi ,n},\;\gamma_{\phi ,n},\;\mathrm{scnb},\;\mathrm{scee},\;A_{ij},\;B_{ij}$ are parameters that are optimized for each force field (see also Section 2.6 below). ϵ represents the relative permittivity; presently, the relative permittivity in the MD calculations and parametric fitting was set to 1.0.

Below, the average over all 31,899 REE datasets is denoted by 〈REE〉.

### 2.5. Calculation of the Restrained Electrostatic Potential Charge

In the GLYCAM06 force field and ff14SB force field models, the restrained electrostatic potential (RESP) charge [13], calculated to reproduce the electrostatic potential, was constant. In the GLYCAM06 force field model, the ensemble average of these RESP charges was used as a fixed atomic charge. However, in the present study, the atomic charge was also subject to the parametric fitting. First, according to the calculation method of the RESP charge in the GLYCAM06 force field model [29], the RESP charge was calculated for each of the 31,899 structures at the HF/6-31G* level by constraining the atomic charges on all of the hydrogen atoms to zero. Then, ensemble average and standard deviation for each atomic charge were calculated.

These ensemble average and standard deviation values were incorporated into the cost function in the present study, so that the fitted values of individual atomic charges did not significantly deviate from ensemble-averaged RESP charges.

### 2.6. Optimization of Force Field Parameters

The structures included in the dataset in Section 3.3 below were sampled to ensure diversity, but it is practically impossible to completely cover the space of structures. Therefore, to avoid overfitting on the training set, and for maintaining accuracy even with respect to unknown structures, we constructed a cost function that included penalty terms. The dihedral angle and atomic charge parameters were optimized so that the REE was reduced by minimizing this cost function.

In what follows, we describe the optimized parameters. The torsional energy is expressed by the sum of the Fourier series for up to six periods, as shown in Equation (4) below, where the amplitude $V_{\phi ,n}$ and phase angle $\gamma_{\phi ,n}$ in the equation are optimized. On the other hand, the non-bonded electrostatic interaction and the 1–4 electrostatic interaction are defined by Equations (5) and (6), respectively, for which the atomic charges $q_{i}$ and 1–4 electrostatic interaction correction factor scee were optimized. In addition, the fixed point charges of all aliphatic hydrogen atoms were set to zero as in the GLYCAM06 force field framework; in this study, the atomic charges of 17 atoms excluding the aliphatic hydrogen atoms were optimized:

(4) $$\begin{array}{cc} E_{\mathrm{TOR}}\left(s,d,\mathrm{FF}\right) & =\sum_{n}^{6}V_{\phi ,n}\left(1+\cos\left(n\phi -\gamma_{\phi ,n}\right)\right), \end{array}$$

(5) $$\begin{array}{cc} E_{C}\left(s,i,j,\mathrm{FF}\right) & =\frac{q_{i}q_{j}}{\epsilon r_{ij}}, \end{array}$$

(6) $$\begin{array}{cc} E_{C14}\left(s,i,j,\mathrm{FF}\right) & =\frac{1}{\mathrm{scee}}\frac{q_{i}q_{j}}{\epsilon r_{ij}}. \end{array}$$

In Equation (4), *s* refers to the sample structure, *d* represents the set of four atoms that are directly bonded, $V_{\phi ,n}$ is the barrier energy of the dihedral angle parameter and ϕ is the dihedral angle of the four atoms. In Equations (5) and (6), *i* and *j* correspond to the atomic pair with the electrostatic interaction, and $r_{ij}$ is the distance between atoms *i* and *j* in structure *s*. The variable *q* represents the atomic charges, while $q_{i},q_{j}$, and scee are defined for each force field. These equations of (4)–(6) correspond to the dihedral angle term, the non-bonded electrostatic interaction term, and the 1–4 electrostatic interaction term in Equation (3), respectively.

The aim of this study is to optimize the force field parameters so as to reduce the relative energy error expressed by Equation (2). However, as a result of optimization without including the penalty term, the barrier energy $V_{\phi ,n}$ of the dihedral angle parameter tends to show too large a value. We supposed that this was due to overlearning, so we introduced the penalty term as shown in Equation (7), whose details are shown below. We then defined cost function *C* as in Equation (7), and optimized it for minimizing REE in Equation (2):

(7) $$C=\frac{1}{M}\sum_{s}^{M}\mathrm{REE}{\left(s\right)}^{2}+\lambda_{\mathrm{csum}}\chi_{\mathrm{csum}}^{2}+\lambda_{\mathrm{chg}}\chi_{\mathrm{chg}}^{2}+\lambda_{\mathrm{dih}}\chi_{\mathrm{dih}}^{2}+\lambda_{\mathrm{scee}}\chi_{\mathrm{scee}}^{2}.$$

Here, *M* represents the total number of sample structures to be used as input, $\chi_{\mathrm{csum}}$ represents the penalty for misalignment with respect to the neutral state of the molecule, i.e., zero total charge, $\chi_{\mathrm{chg}}$ represents the penalty for misalignment with respect to the average RESP charge of each atom, $\chi_{\mathrm{dih}}$ represents the penalty for deviation from the barrier energy of the GLYCAM06 force field, and $\chi_{\mathrm{scee}}$ represents the penalty for deviation from the scaling factor of the 1–4 electrostatic interaction of the GLYCAM06 force field. $\lambda_{\mathrm{csum}},\lambda_{\mathrm{chg}},\lambda_{\mathrm{dih}}$, and $\lambda_{\mathrm{scee}}$ are hyper-parameters that correspond to the weight coefficients of the total charge, the charge of each atom, the barrier energy of the dihedral angle, and the scaling factor penalties for the 1–4 electrostatic interactions. In the present study, we tuned the hyper-parameters for minimizing $\mathrm{REE}{\left(s\right)}^{2}$ with s∈test over the test set. In addition, the system must be neutral when the periodic boundary condition is used, where the target molecule, β-glucose, is a neutral molecule; therefore, $\lambda_{\mathrm{csum}}$ was set to a high value, $10^{7}$, for constraining the total charge to zero. Other hyper-parameters, $\lambda_{\mathrm{chg}},\lambda_{\mathrm{dih}}$ and $\lambda_{\mathrm{scee}}$ were set to 0.1, 3.0, 100.0, respectively. These penalties were defined, respectively, by Equations (8)–(11):

(8) $$\begin{array}{cc} \chi_{\mathrm{csum}}^{2} & ={\left(\sum_{i}q_{i}\right)}^{2}, \end{array}$$

(9) $$\begin{array}{cc} \chi_{\mathrm{chg}}^{2} & =\sum_{i}\frac{{\left(q_{i}-\bar{q_{i}}\right)}^{2}}{{\left(2\sigma_{i}\right)}^{2}}, \end{array}$$

(10) $$\begin{array}{cc} \chi_{\mathrm{dih}}^{2} & =\sum_{\phi \in \mathrm{dihedrals}}\sum_{n}^{6}{\left(V_{\phi ,n}^{\mathrm{OPT}}-V_{\phi ,n}^{\mathrm{GLYCAM}06}\right)}^{2}, \end{array}$$

(11) $$\begin{array}{cc} \chi_{\mathrm{scee}}^{2} & ={\left({\mathrm{scee}}^{\mathrm{OPT}}-{\mathrm{scee}}^{\mathrm{GLYCAM}06}\right)}^{2}. \end{array}$$

In Equations (8) and (9), $q_{i}$ represents the atomic charge, where the subscript *i* represents the 17 atoms subject to the optimization. $\bar{q_{i}}$ and $\sigma_{i}$ in Equation (9) are the average and standard deviation of RESP charges, calculated for all 31,899 structures. $V_{\phi ,n}^{\mathrm{OPT}}$ and $V_{\phi ,n}^{\mathrm{GLYCAM}06}$ in Equation (10) represent the barrier energies of the dihedral angle parameter after parametric fitting and in the GLYCAM06 force field, respectively. ${\mathrm{scee}}^{\mathrm{OPT}}$ and ${\mathrm{scee}}^{\mathrm{GLYCAM}06}$ in Equation (11) represent the 1–4 electrostatic interaction scaling factors after parametric fitting and in the GLYMCAM06 force field, respectively.

The cost function in Equation (7) was minimized using the Adadelta algorithm [30], which is an improved gradient descent method. The entire dataset was divided into a training set and a test set in the ratio of 7:3 for cross-validation.

The implemented methods used the TensorFlow library [31] and were executed in Python. The code used for parametric optimization in this work has been uploaded to the GitHub server: https://github.com/mmikejo/optimization_force_field_parameters_for_carbohydrate (accessed on 3 November 2021). Details about the code are given in Supplementary Materials (SM). The optimized parameters are listed in Table 1 below and in Table S1 in Supplementary Materials.

### 2.7. Evaluation of Optimized Parameters

For assessing the parameters optimized using the above method, the 〈REE〉 improvement metric $\Delta_{d}$, capturing the changes due to the dihedral angle parameter values related to the dihedral angle set *d*, and the 〈REE〉 improvement metric $\Delta_{i}$, capturing the changes associated with the atomic charge $q_{i}$, were defined as follows:

(12) $$\begin{array}{c} \begin{array}{cc} \Delta_{d} & =\frac{1}{M}\sum_{s}^{M}(E_{\mathrm{TOR}}\left(s,d,\mathrm{GLYCAM}06\right)-E_{\mathrm{TOR}}\left(s,d,\mathrm{OPT}\right)), \end{array} \end{array}$$

(13) $$\begin{array}{c} \begin{array}{cc} \Delta_{i} & =\frac{1}{M}\sum_{s}^{M}\{\frac{1}{2}\sum_{j}(E_{C}\left(s,i,j,\mathrm{GLYCAM}06\right)-E_{C}\left(s,i,j,\mathrm{OPT}\right))\\ \; & +\frac{1}{2}\sum_{j^{\prime }}(E_{C14}\left(s,i,j^{\prime },\mathrm{GLYCAM}06\right)-E_{C14}\left(s,i,j^{\prime },\mathrm{OPT}\right))\}. \end{array} \end{array}$$

Here, OPT represents our proposed (optimized) force field.

We further defined the sum of the improvement metric in 〈REE〉 by modifying the dihedral angle and atomic charge parameters for all atoms, as $\Delta_{\mathrm{DIH}}$ and $\Delta_{\mathrm{CHG}}$, respectively:

(14) $$\begin{array}{c} \Delta_{\mathrm{DIH}}=\sum_{d}\Delta_{d}, \end{array}$$

(15) $$\begin{array}{c} \Delta_{\mathrm{CHG}}=\sum_{i}\Delta_{i}. \end{array}$$

In this case, the improvement metric was the sum of the first power rather than that of squares to ensure that 〈REE〉 (which we sought to minimize presently) has the following additivity property:

(16) $${\langle \mathrm{REE}\rangle }^{\mathrm{GLYCAM}06}-{\langle \mathrm{REE}\rangle }^{\mathrm{OPT}}=\Delta_{\mathrm{DIH}}+\Delta_{\mathrm{CHG}}.$$

We explored the dihedral angle and atomic charge parameters that importantly contributed to improving 〈REE〉 by calculating the improvement metric values $\Delta_{d}$ and $\Delta_{i}$.

### 2.8. Comparative Analysis of Force Fields by MD Simulations

MD simulations were performed using the GLYCAM06 force field and the present force field for comparative analysis and further validation of the optimized parameters. The system was the same as the one that was used for structural sampling in Section 2.1 except that the cutoff radius was set to 9.0 Å. Under the periodic boundary condition, sampling of 5000 structures was performed in the 50-ns-long MD runs after 2-ns-long relaxation, where we employed NPT ensemble at the temperature of 300 K and pressure of 1 bar.

The obtained trajectory suggests that the dihedral angles strongly contribute to improving REE given by Equation (2). The Cremer-Pople (CP) puckering parameters [32], describing the relationship of the six-membered ring structure of monosaccharides, were then compared; subsequently, analysis was conducted, focusing on the properties on which the distributions differed greatly between the two force fields.

Two analytical methods were employed: (1) comparison with NMR measurements and (2) comparison of energy dependence on structural deformations. For comparison with NMR measurements, we used a report [24] on the NMR measurements of free β-glucose, in which the hydroxyl group was not modified. The literature values and the distributions obtained from the MD simulations of the $O_{5}$–$C_{5}$–$C_{6}$–$O_{6}$ dihedral angle were compared. In addition, the energy dependence was compared for QM and MM calculations with respect to structural deformations, in which we confirmed different trends for the MD simulations using the GLYCAM06 and our proposed force fields. The root mean square error (RMSE) was defined as in Equation (17) below, using the relative energy error given by Equation (2), and thus we compared the presently computed force field with the GLYCAM06 force field.

(17) $$\mathrm{RMSE}=\sqrt{\frac{1}{M}\sum_{s}^{M}{\mathrm{REE}(s)}^{2}}.$$

The deformation modes examined in this study were rotations around the $C_{5}$–$C_{6}$ and $O_{1}$–$C_{1}$ bonds, and the ring flip of the $C_{1}$ carbon of the six-membered ring. For structure validation, 20 structures of the ${}^{4}C_{1}$ chair conformation were randomly selected from the test set; note that these structures were not used for parametric fitting. For the rotation validation, 36 structures, with angles ranging from 0${}^{\circ }$(degrees) to 350${}^{\circ }$, were created with angular steps of 10${}^{\circ }$. On the other hand, for ring flipping validation, 10 structures were considered with angles ranging from −4.0${}^{\circ }$ to 5.0${}^{\circ }$ and the angular steps of 1.0${}^{\circ }$ based on the original structure. The reference structure that was used for calculating the relative energy error was the most stable structure in the QM calculations for each deformation. The level of the QM calculations was B3LYP/6-31++G(2d,2p), the same as the one that was used for calculating the relative energy error in Section 2.4.

## 3. Results and Discussion

### 3.1. Structure Optimization

**Figure 2:**
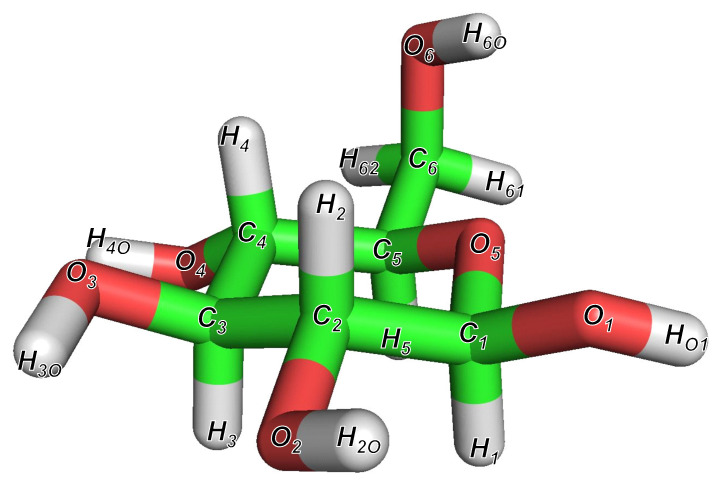
The most stable structure of β-glucose at B3LYP/6-31++G(2d,2p) DFT computation level. This structure has the same characteristics as the most stable structure reported by Alonso et al. [33].

The most stable structure of β-glucose obtained after structural optimization using the QM calculations is shown in Figure 2. The features of this structure are consistent with those of the most stable structure described in the structural analysis report of β-glucose by Alonso et al. [33]. In addition, the sampling structures obtained using the MD simulations at 300 K and 600 K are shown for the system of C-P puckering coordinates in Figures S2 and S3 in Supplementary Materials (SM).

### 3.2. RESP Charge Distribution

The RESP charge distribution for the atoms in the 31,899 structures was unimodal. There was a difference in the spread of the distribution depending on the atoms. The results are shown in Figure S4 in SM.

### 3.3. Optimization and Evaluation of Force Field Parameters

Table 1 lists the resulting atomic charges, while Table S1 in SM lists the resulting dihedral angle parameters. The force field parameter files (Prep and frcmod files for β-glucose) optimized in this study are also available from the GitHub server: https://github.com/mmikejo/optimization_force_field_parameters_for_carbohydrate (accessed on 3 November 2021). The scaling factor scee for the 1–4 electrostatic interaction obtained by parametric optimization is 1.017.

Table 2 lists 〈REE〉 for the GLYCAM06 system and for the present force field framework.

**Figure 3:**
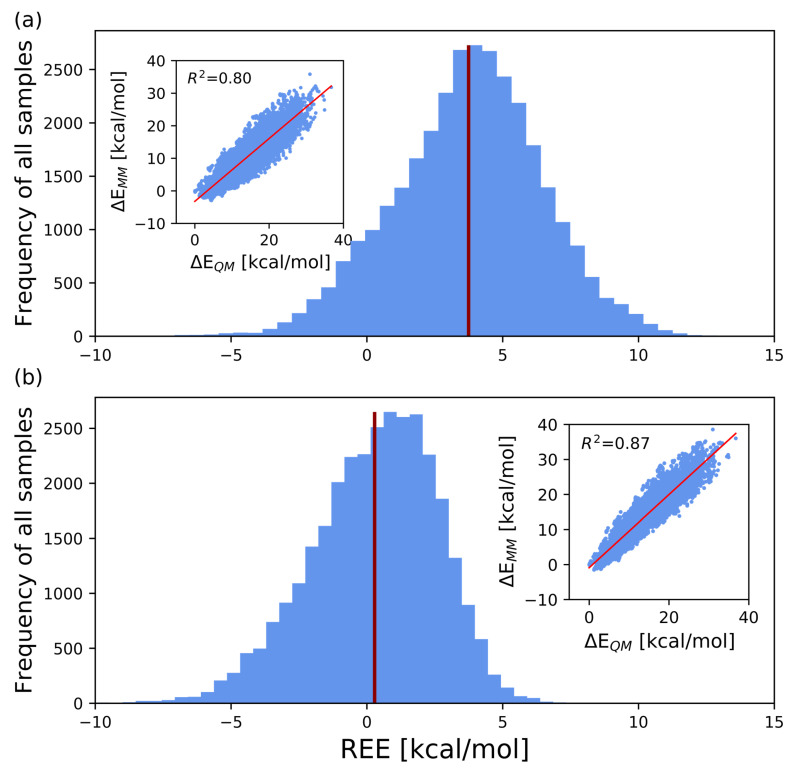
Histogram of REEs for all 31,899 structures, with REE on the abscissa and the number of samples on the ordinate. The red bars indicate the average values of REE. The scatter diagram in the inset is also plotted with $\Delta E_{\mathrm{QM}}$ on the abscissa and $\Delta E_{\mathrm{MM}}$ on the ordinate. (**a**,**b**), respectively, show the results obtained using the GLYCAM06 force field framework and our proposed force field framework for computing the MM energies.

After optimizing the force field parameters, the structure average of REE changed from 3.744 kcal/mol to 0.282 kcal/mol, thus showing a significant improvement. The standard deviation decreased, from 2.728 kcal/mol to 2.318 kcal/mol. The table also shows the respective contributions of the optimized dihedral angle and atomic charge parameters to 〈REE〉. The extent of improvement, $\Delta_{\mathrm{DIH}}$ (see Equation (14)), due to the modification of the dihedral angle parameters, was 2.681 kcal/mol. On the other hand, $\Delta_{\mathrm{CHG}}$ (see Equation (15)) owing to the modification of atomic charges and the 1–4 electrostatic interaction, the scaling factor scee was 0.781 kcal/mol. Figure 3 shows the distributions of REEs for the GLYCAM06 framework and for the force field framework proposed in this work.

**Figure 4:**
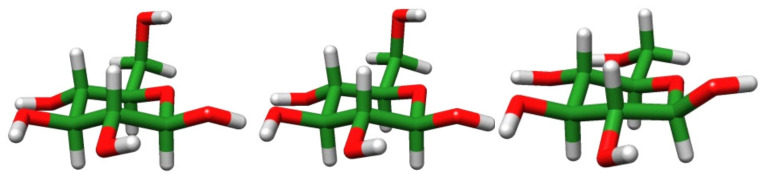
From left to right: the most stable structures obtained by DFT calculation, MM calculations using our proposed force field framework, and using the GLYCAM06 force field framework, respectively.

The most stable structures for each calculation method are shown in Figure 4. Using the GLYCAM06 force field framework, the most stable conformation was Tg+/cc/t, whereas using the QM calculations and our proposed force field framework, the most stable conformation was G-g+/cc/t, consistent with the experimental work [33]. The definition of this structure is the same as in the previous study [33], and the details are provided in Section SM-5 of SM.

The iterative behaviors of cost function for the training and test sets are shown in Figure S5 in SM, and the scatter plots of $\Delta E_{\mathrm{QM}}=E_{\mathrm{QM},i}-E_{\mathrm{QM},\mathrm{ref}}$ versus $\Delta E_{\mathrm{MM}}=E_{\mathrm{MM},i}-E_{\mathrm{MM},\mathrm{ref}}$ and the REE distributions for the training and test sets are shown in Figure S6 in SM. Evidently, no overfitting occurred during the parametric optimization.

Some local structures that seem to be important for improving energy are discussed below, including the cases in which the improvement by the present force field does not always work perfectly. We emphasize here that the parametric fitting in this study improved the GLYCAM06 force field by slightly and accumulatively improving the MM energies of the respective local structures.

#### 3.3.1. Hydroxymethyl Group at $C_{6}$

**Figure 5:**
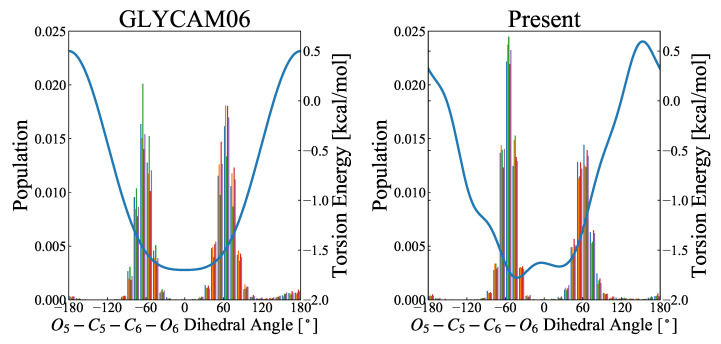
Distribution of $O_{5}$–$C_{5}$–$C_{6}$–$O_{6}$ rotamers by MD simulations. The abscissa refers to the dihedral angle ranging from −180${}^{\circ }$ to 180${}^{\circ }$, and the ordinate is the probability density function calculated over 5000 snapshots. The left and right figures, respectively, show the results for the GLYCAM06 force field and the force field proposed herein as the molecular force field for the MD simulations. The MD calculations were performed for 50-ns-long product runs in five replicates (represented by colors), and the rotamer distribution was calculated from each of the 5000 snapshots. The fractions of gg, gt, and tg were, respectively, 45.4%, 51.7%, and 2.8% for the GLYCAM06 force field, and 58.0%, 40.5%, and 1.6% for the force field proposed herein. The blue curve in each figure represents the potential energy of torsional motion ($O_{5}$–$C_{5}$–$C_{6}$–$O_{6}$) associated with a change in the dihedral angle of $O_{5}$–$C_{5}$–$C_{6}$–$O_{6}$.

The population distribution of the rotamer at the 6-hydroxymethyl group was compared using the trajectories obtained in the MD simulations, for the GLYCAM06 and the force fields proposed here. For the GLYCAM06 force field, the gg:gt ratio was 0.45:0.52, whereas for the force field proposed here, it was 0.58:0.41; that is, gg appeared favorably (see Figure 5). Thus, the result obtained for our proposed force field well reproduces the experimental result [24], where the gg:gt ratio was found to be 0.60:0.40. This is because the dihedral angle parameter of $O_{5}$–$C_{5}$–$C_{6}$–$O_{6}$ is optimized and the torsional energy changes, as shown in Figure 5.

**Figure 6:**
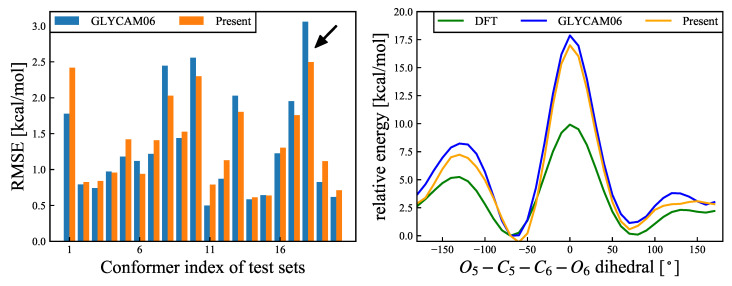
(**Left**): RMSEs (Equation (17)) for $C_{5}$–$C_{6}$ rotational deformation modes for each of the 20 structures. Blue and orange bars refer to the results calculated using the GLYCAM06 force field and the force field proposed herin, respectively. The black arrow refers to the structure with the most improved RMSE, and the energy change of this structure is shown in the right panel. The average RMSE values for the 20 structures were 1.327 kcal/mol for the GLYCAM06 force field and 1.351 kcal/mol for the our proposed force field. (**Right**): Relative energy ($E_{s}-E_{\mathrm{ref}}$) changes for the structure with the most improved RMSE as an example. The abscissa represents the $O_{5}$–$C_{5}$–$C_{6}$–$O_{6}$ dihedral angle, and the ordinate represents the relative energy for the DFT and MM calculations, for the two different force fields. The reference structure was the most stable structure for the DFT calculations. The green, blue, and orange curves represent the relative energy changes for the DFT calculations, the GLYCAM06 force field, and our proposed force field, respectively.

The torsional energies of the GLYCAM06 force field are equivalent between at $-60^{\circ }$ and $60^{\circ }$, and the chirality of asymmetric carbon $C_{5}$ is not considered, likely because the chirality of the model molecule, 2-methoxypropanol, was not taken into account when creating the GLYCAM06 force field. To verify this issue, the energy change with rotation around $C_{5}$–$C_{6}$ was compared between the QM and MM calculations. The results are presented in Figure 6. Of the 20 structures (see Section 2.8 above) used for examination, for six structures the RMSE decreased by 0.1 kcal/mol or more for our proposed force field, while for six other structures the RMSE increased. It was also confirmed that the average RMSE of the 20 structures was 1.327 kcal/mol for the GLYCAM06 force field and 1.351 kcal/mol for our proposed force field; that is, the RMSE was slightly higher for our proposed force field in this case, while the difference was minor. This is because the energy change accompanying the rotation of the hydroxymethyl group at $C_{6}$ is influenced not only by the dihedral angle parameter of the $C_{5}$–$C_{6}$ rotation but also by the orientation of the hydroxyl group at $C_{6}$ and $C_{4}$.

#### 3.3.2. Hydroxyl Group at $C_{1}$

**Figure 7:**
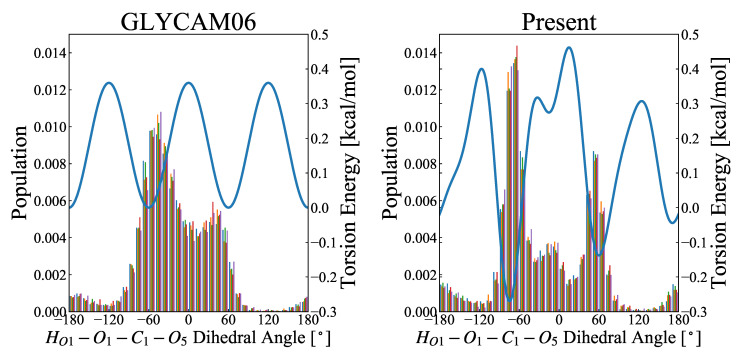
Distribution of $H_{O1}$–$O_{1}$–$C_{1}$–$O_{6}$ rotamers, obtained in the MD simulations. The abscissa represents the dihedral angle, ranging from −180${}^{\circ }$ to 180${}^{\circ }$, and the ordinate is the probability density function calculated over 5000 snapshots. The left and right figures, respectively, show the results for the GLYCAM06 force field and our proposed force field as the molecular force fields for the MD simulations. The MD calculations were performed for 50-ns-long product runs in five replicates (represented by colors), and the rotamer distribution was calculated from each of the 5000 snapshots. The blue curve in each panel represents the potential energy for torsional motion ($H_{O1}$–$O_{1}$–$C_{1}$–$O_{6}$) associated with a change in the dihedral angle of $H_{O1}$–$O_{1}$–$C_{1}$–$O_{6}$.

Similarly, the population distribution of the rotamer at the 1-hydroxymethyl group was compared in terms of the trajectories obtained by the MD simulations, for GLYCAM06 and presently proposed force fields. For both of the force fields, peaks appeared at −60${}^{\circ }$ and 60${}^{\circ }$, but there was a significant difference between the two frameworks regarding the appearance rate at 0${}^{\circ }$ (Figure 7).

**Figure 8:**
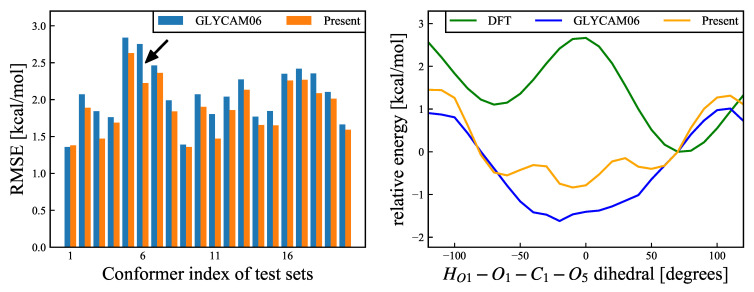
(**Left**): RMSEs (Equation (17)) for $O_{1}$–$C_{1}$ rotational deformation modes, for each of the 20 structures. Blue and orange bars refer to the results calculated using the GLYCAM06 force field and our proposed force field, respectively. The black arrow refers to the structure with the most improved RMSE, and the energy change of this structure is shown in the right panel. The average RMSE values for the 20 structures were 2.058 kcal/mol for the GLYCAM06 force field and 1.886 kcal/mol for the present force field. (**Right**): Relative energy change for the structure with the most improved RMSE as an example. The abscissa shows the $H_{O1}$–$O_{1}$–$C_{1}$–$O_{5}$ dihedral angle, and the ordinate shows the relative energies for the DFT calculations and MM calculations, for the two different force fields. The reference structure was the most stable structure according to the DFT calculations. The green, blue, and orange curves show the relative energies for the DFT calculations, the GLYCAM06 force field, and our proposed force field, respectively.

Since no experimental results (e.g., NMR-based ones) have been reported to support these results, the rotamer energy has been validated using the QM calculations. The results are shown in Figure 8. The validation results suggest that, based on the QM calculations, one of the rotamers of $-60^{\circ }$ and $60^{\circ }$ is the most stable structure, and the other is the local stable structure; the energy is destabilized near $0^{\circ }$ in between. On the other hand, based on the MM calculations, the stabilization is optimal near $0^{\circ }$. This is because the attractive force owing to the 1–4 electrostatic interaction between the positively charged $H_{O1}$ atom and the negatively charged $O_{5}$ atom is the strongest near $0^{\circ }$, where the distance between the two atoms is minimal. At the same time, the torsional energy of $H_{O1}$–$O_{1}$–$C_{1}$–$O_{5}$ is minimal near $-60^{\circ }$ and $60^{\circ }$ and maximal near $0^{\circ }$. The rotamer energy of the 1-hydroxyl group is governed by the balance between the 1–4 electrostatic interactions and torsional energy. Compared with the GLYCAM06 force field framework, the RMSE is smaller for our proposed force field framework, owing to the weaker 1–4 electrostatic interactions and the higher torsional energy; thus, the rotamer population at the 1-hydroxymethyl group around $0^{\circ }$ for the MD simulations is smaller. Of the 20 structures used for validation, 14 structures exhibited the RMSE reduction by more than 0.1 kcal/mol, while the remaining six structures showed no significant change in the RMSE. The mean RMSE over the 20 structures was 2.058 kcal/mol for the GLYMCAM06 force field framework, and 1.886 kcal/mol for our proposed force field framework, respectively, confirming the improvement owing to the parametric fitting. However, while the discrepancy in the energy change owing to the rotation of the 1-hydroxyl group was slightly improved by the parametric fitting, the energy change owing to the structural change for the MM calculations did not completely agree with that for the QM calculations; thus, there is room for further improvement.

#### 3.3.3. Six-Membered Ring Flip

**Figure 9:**
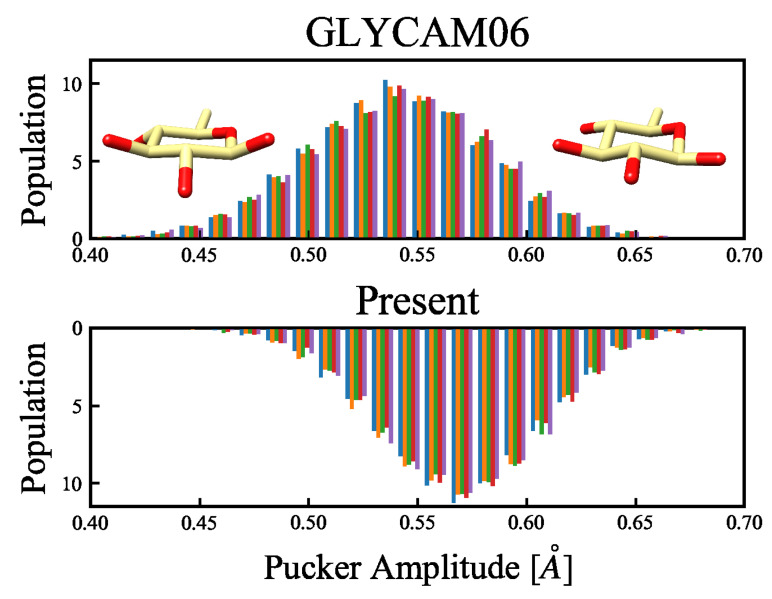
Distribution of the amplitude of the CP puckering parameters, according to the MD simulations. The abscissa shows the puckering amplitude, while the ordinate is the probability density function, calculated over 5000 snapshots. The upper and lower panels, respectively, show the results obtained using the GLYCAM06 force field and our proposed force field as the molecular force fields for the MD simulations. The MD calculations were performed for 50-ns-long product runs in five replicates (represented by colors), and the puckering amplitude was calculated from each of the 5000 snapshots. The structures in the inset show, from left to right, a flat ring with a small amplitude, and an uneven ring with a large amplitude.

The MD simulations at 300 K confirmed a significant difference between the ratios of the CP-puckering coordinates. The results are shown in Figure 9.

**Figure 10:**
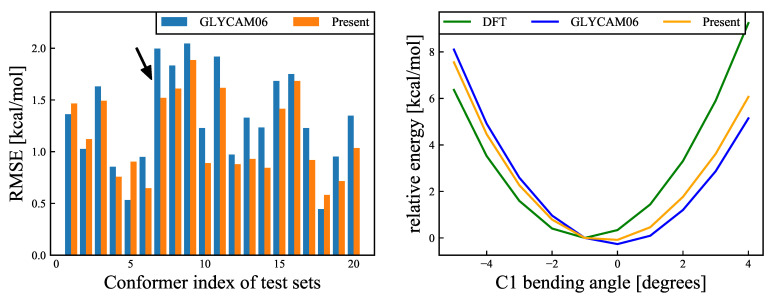
(**Left**): RMSEs (Equation (17)) for ring-flip deformation modes, for each of the 20 structures. Blue and orange bars refer, respectively, to the results calculated using the GLYCAM06 force field and our proposed force field. The black arrow refers to the structure with the most improved RMSE, and the energy change of this structure is shown in the right panel. The average RMSE values for the 20 structures were 1.315 kcal/mol for the GLYCAM06 force field and 1.145 kcal/mol for our proposed force field. (**Right**): Relative energy variations for the structures with the most improved RMSE as an example. The abscissa shows the $C_{1}$ bending angle, and the ordinate shows the relative energy for the DFT calculations and MM calculations, for the two different force fields. The reference structure was the most stable structure according to the DFT calculations. The green, blue, and orange curves show, respectively, the relative energy variations for the DFT calculation, the GLYCAM06 force field, and our proposed force field.

The six-membered ring structures that were sampled in the MD simulations at 300 K all had ${}^{4}C_{1}$ chair conformations, where structures with small amplitudes corresponded to flat-ring structures, whereas structures with larger amplitudes corresponded to uneven-ring structures (see the insets of Figure 9). In an earlier report by Mayes et al. [34], the puckering amplitude of the most stable structure of β-glucose calculated using the first-principles approach under gas-phase conditions was 0.56 Å, suggesting that the modification of the force field parameters in the present study facilitated the emergence of stable structures. Based on this, we confirmed that the energy change owing to the structural deformation of the ring flip was improved. This ring flip is an interconversion between the cyclic conformers caused by the rotation around a single bond. Considering that Barnett et al. [35] reported, based on their QM/MM calculations, that a cyclic conformer change is important for substrates in enzymatic reactions, six-membered cyclic conformers of monosaccharides seem to be important for accurate force field determination. Comparison of the energy changes related to the $C_{1}$ carbon flip across the QM calculations and the MM calculations with the GLYCAM06 force field and our proposed force field are shown in Figure 10. This comparison reveals that the average RMSE of the 20 structures is 1.315 kcal/mol for the GLYCAM06 force field and 1.145 kcal/mol for the optimized force field.

#### 3.3.4. Effect of Atomic Charges

In the atomic charge parametric optimization, the modification of the charge parameters of the 1-hydroxyl group contributed significantly to the improvement of 〈REE〉. The reason for this is that the constraint on the atomic charge parameters of the 1-hydroxyl group was removed in the GLYCAM06 force field framework. Moreover, the modification of the atomic charges of functional groups susceptible to the atomic charges of the 1-hydroxyl group, such as the 6-hydroxyl group, 2-hydroxyl group and $O_{5}$, was important for reducing 〈REE〉.

#### 3.3.5. Remark on the Lennard-Jones Potential Parameters

We did not fit the Lennard-Jones potential parameters in the present parametric fitting study. There are two reasons for this: (1) the energy estimated from the QM calculations for one β-glucose molecule in vacuum was used as a reference value for the fitting process, and (2) the accuracy of the QM calculations in this case was similar to that of the GLYCAM06 force field because there was no dispersion correction. However, as described in Section 2.3, the structure for which the Lennard-Jones potential was overestimated in the MM calculations was actually found; therefore, the possibility of fitting the Lennard-Jones potential parameters may also be discussed in the future research. Concerning the charge parameters and the Lennard-Jones potential parameters related to the non-bonding interactions, only the charge parameters were fitted, which was not considered to bring about a problem since previous studies [29,36] also fitted only the atomic charge parameters.

## 4. Conclusions

In this study, as a re-optimization of the GLYCAM06 force field used in the AMBER package, parametric fitting of the dihedral angles and atomic charges was performed to reproduce the relative energy difference with respect to QM calculations for β-glucose. This novel parametric fitting reduced the relative energy error 〈REE〉 from 3.744 kcal/mol in the GLYCAM06 force field framework to 0.282 kcal/mol. To examine the accuracy of the proposed force field, MD simulations were performed using the GLYCAM06 force field framework and the novel proposed force field framework, for comparative analysis. Our validation analysis confirmed that the distributions of rotamers of the hydroxymethyl group at the 6-position, for which the experimental results were reported, were better reproduced. In addition, it was also confirmed that other structural deformations were reasonably described, compared with the QM calculations. We believe that the method used here can improve the force field estimation for glycans more extensively, by advancing the fitting of the monosaccharide parameters other than the β-glucose and the dihedral angle parameters of glycosidic bonds. Research in this direction is currently underway.

## Figures and Tables

**Table 1:** Optimized atomic partial charges, excluding all aliphatic hydrogen atoms, for β-glucose.

Atomic Label	Atomic Charge ^a^	Atomic Label	Atomic Charge ^a^	Atomic Label	Atomic Charge ^a^
$C_{1}$	0.3807	$H_{O1}$	0.4278	$O_{1}$	−0.6496
$C_{2}$	0.3003	$O_{2}$	−0.6958	$H_{2O}$	0.4496
$C_{3}$	0.2904	$O_{3}$	−0.7011	$H_{3O}$	0.4365
$C_{4}$	0.2602	$O_{4}$	−0.7168	$H_{4O}$	0.4256
$C_{5}$	0.2169	$O_{5}$	−0.4806	$C_{6}$	0.2861
$O_{6}$	−0.6717	$H_{6O}$	0.4413		

^a^ In units of elementary charge *e*.

**Table 2:** Improvement of 〈REE〉, Δ, for two parameter sets of GLYCAM06 and the present force fields.

Force Field	〈REE〉 [kcal/mol]	Δ [kcal/mol] ^a^
GLYCAM06	3.744	
Charge and dihedral angle parameters in the present work	0.282	3.463
Only charge parameters in the present work	2.964	0.781
Only dihedral angle parameters in the present work	1.064	2.681

^a^ see Equations (14)–(16).

## Data Availability

The data that support the findings of this study and are not found in Supplementary Materials are available from the corresponding authors upon reasonable request.

## Supplementary Materials

The following are available online. Figure S1: Mercator projections of the CP puckering coordinates of β-glucose conformers. The red dots represent the typical conformers. Figure S2. Mercator projections of the CP puckering coordinates of structural sampling at 300 K. The blue dots represent the structures obtained by MD, and the red dots represent the conformers shown in Figure S1. Figure S3. Mercator projections of the CP puckering coordinates of structural sampling at 600 K. The blue dots represent the structures obtained by MD, and the red dots represent the conformers shown in Figure S1. Figure S4. Distribution of RESP charges for all 31,899 structures. Atomic labels correspond to Figure 1 in the main text. Figure S5. Evaluation of cost function. The abscissa refers to fitting iterations, and the ordinate is *C* and $\langle {\mathrm{REE}}^{2}\rangle$, where blue and red colors show *C* of Equation (7) for training and test sets, respectively, and light blue and pink colors show $\langle {\mathrm{REE}}^{2}\rangle$ of Equation (7) for training and test sets, respectively. Figure S6. Histogram of REEs for training and test set structures, with REE on the abscissa and the number of samples on the ordinate. The red bars indicate the average values of REE. The scatter diagram in the inset is also plotted with $\Delta E_{QM}$ on the abscissa and $\Delta E_{MM}$ on the ordinate. (a) and (b) show the results obtained using the GLYCAM06 force field for computing the MM energies with training and test set structures, respectively. (c) and (d) show the results obtained using the the presently proposed force field for computing the MM energies with training and test set structures, respectively. Figure S7. Newman projections of the conformations of the hydroxymethyl group around $C_{5}$–$C_{6}$ and $C_{6}$–$O_{6}$ bonds. Figure S8. The clockwise (C) and counter-clockwise (CC) conformers of β-glucose. Figure S9. Newman projections of the conformations of the hydroxyl group around $C_{1}$–$O_{1}$ bonds. Table S1: Torsion parameters in Equation (4) of the main text. The columns headed $V_{1}$ to $V_{6}$ have units of kcal/mol, and the columns headed $\gamma_{1}$ to $\gamma_{6}$ have units of degrees (${}^{\circ }$).

## Sample Availability

Not available.

## Abbreviations

The following abbreviations are used in this manuscript:

MD	molecular dynamics
MM	molecular mechanics
QM	quantum mechanics
DFT	density functional theory
REE	relative energy error
RESP	restrained electrostatic potential

## References

[1] Haltiwanger R.S., Lowe J.B. (2004). Role of Glycosylation in Development. Annu. Rev. Biochem..

[2] Ohtsubo K., Marth J.D. (2006). Glycosylation in Cellular Mechanisms of Health and Disease. Cell.

[3] Viswanathan K., Chandrasekaran A., Srinivasan A., Raman R., Sasisekharan V., Sasisekharan R. (2010). Glycans as receptors for influenza pathogenesis. Glycoconjug. J..

[4] Nagae M., Yamaguchi Y. (2012). Function and 3D Structure of the N-Glycans on Glycoproteins. Int. J. Mol. Sci..

[5] Lenman A., Liaci A.M., Liu Y., Frängsmyr L., Frank M., Blaum B.S., Chai W., Podgorski I.I., Harrach B., Benko M. (2018). Polysialic acid is a cellular receptor for human adenovirus 52. Proc. Natl. Acad. Sci. USA.

[6] Park M.S. (2015). Molecular Dynamics Simulations of the Human Glucose Transporter GLUT1. PLoS ONE.

[7] Xu D., Newhouse E.I., Amaro R.E., Pao H.C., Cheng L.S., Markwick P.R., McCammon J.A., Li W.W., Arzberger P.W. (2009). Distinct Glycan Topology for Avian and Human Sialopentasaccharide Receptor Analogues upon Binding Different Hemagglutinins: A Molecular Dynamics Perspective. J. Mol. Biol..

[8] Siebert H.C., Lieth C.W.V.D., Dong X., Reuter G., Schauer R., Gabius H.J., Vliegenthart J.F. (1996). Molecular dynamics-derived conformation and intramolecular interaction analysis of the N-acetyl-9-O-acetylneuraminic acid-containing ganglioside GD1a and NMR-based analysis of its binding to a human polyclonal inununoglobulin G fraction with selectivity for O-acetylated sialic acids. Glycobiology.

[9] Yoda T., Sugita Y., Okamoto Y. (2004). Secondary-structure preferences of force fields for proteins evaluated by generalized-ensemble simulations. Chem. Phys..

[10] Pérez S., Imberty A., Engelsen S.B., Gruza J., Mazeau K., Jimenez-Barbero J., Poveda A., Espinosa J.F., van Eyck B.P., Johnson G. (1998). A comparison and chemometric analysis of several molecular mechanics force fields and parameter sets applied to carbohydrates. Carbohydr. Res..

[11] Spiwok V., Králová B., Tvaroška I. (2010). Modelling of b-d-glucopyranose ring distortion in different force fields: A metadynamics study. Carbohydr. Res..

[12] Mortier W.J., Ghosh S.K., Shankar S. (1986). Electronegativity-equalization method for the calculation of atomic charges in molecules. J. Am. Chem. Soc..

[13] Bayly C.I., Cieplak P., Cornell W.D., Kollman P.A. (1993). A well-behaved electrostatic potential based method using charge restraints for deriving atomic charges: The RESP model. J. Phys. Chem..

[14] Lamoureux G., Harder E., Vorobyov I.V., Roux B., MacKerell A.D. (2006). A polarizable model of water for molecular dynamics simulations of biomolecules. Chem. Phys. Lett..

[15] Buck M., Bouguet-Bonnet S., Pastor R.W., MacKerell A.D. (2006). Importance of the CMAP Correction to the CHARMM22 Protein Force Field: Dynamics of Hen Lysozyme. Biophys. J..

[16] Maier J.A., Martinez C., Kasavajhala K., Wickstrom L., Hauser K.E., Simmerling C. (2015). ff14SB: Improving the Accuracy of Protein Side Chain and Backbone Parameters from ff99SB. J. Chem. Theory Comput..

[17] Case D., Babin V., Berryman J., Betz R., Cai Q., Cerutti D., Cheatham T., Darden T., Duke R., Gohlke H. (2014). AMBER 14, University of California, San Francisco.

[18] Case D., Betz R., Cerutti D., Cheatham T., Darden T., Duke R., Giese T., Gohlke H., Götz A., Homeyer N. (2016). Amber 16, University of California, San Francisco.

[19] Li Y., Li H., Pickard F.C., Narayanan B., Sen F.G., Chan M.K., Sankaranarayanan S.K., Brooks B.R., Roux B. (2017). Machine Learning Force Field Parameters from Ab Initio Data. J. Chem. Theory Comput..

[20] Kato K., Masuda T., Watanabe C., Miyagawa N., Mizouchi H., Nagase S., Kamisaka K., Oshima K., Ono S., Ueda H. (2020). High-Precision Atomic Charge Prediction for Protein Systems Using Fragment Molecular Orbital Calculation and Machine Learning. J. Chem. Inf. Model..

[21] Kruglov I., Sergeev O., Yanilkin A., Oganov A.R. (2017). Energy-free machine learning force field for aluminum. Sci. Rep..

[22] Kirschner K.N., Yongye A.B., Tschampel S.M., González-Outeiriño J., Daniels C.R., Foley B.L., Woods R.J. (2008). GLYCAM06: A generalizable biomolecular force field. Carbohydrates. J. Comput. Chem..

[23] Marianski M., Supady A., Ingram T., Schneider M., Baldauf C. (2016). Assessing the Accuracy of Across-the-Scale Methods for Predicting Carbohydrate Conformational Energies for the Examples of Glucose and a-Maltose. J. Chem. Theory Comput..

[24] Nishida Y., Hori H., Ohrui H., Meguro H. (1988). ^1^H NMR analyses of rotameric distribution of C5-C6 bonds of d-glucopyranoses in solution. J. Carbohydr. Chem..

[25] Frisch M.J., Trucks G.W., Schlegel H.B., Scuseria G.E., Robb M.A., Cheeseman J.R., Scalmani G., Barone V., Mennucci B., Petersson G.A. (2009). Gaussian 09, Revision A.02.

[26] Frisch M.J., Trucks G.W., Schlegel H.B., Scuseria G.E., Robb M.A., Cheeseman J.R., Scalmani G., Barone V., Petersson G.A., Nakatsuji H. (2016). Gaussian 16, Revision C.01.

[27] Essmann U., Perera L., Berkowitz M.L., Darden T., Lee H., Pedersen L.G. (1995). A smooth particle mesh Ewald method. J. Chem. Phys..

[28] Jorgensen W.L., Chandrasekhar J., Madura J.D. (1983). Comparison of simple potential functions for simulating liquid water. J. Chem. Phys..

[29] Basma M., Sundara S., Çalgan D., Vernali T., Woods R.J. (2001). Solvated ensemble averaging in the calculation of partial atomic charges. J. Comput. Chem..

[30] Zeiler M.D. (2012). ADADELTA: An Adaptive Learning Rate Method. arXiv.

[31] Abadi M., Agarwal A., Barham P., Brevdo E., Chen Z., Citro C., Corrado G.S., Davis A., Dean J., Devin M. (2015). TensorFlow: Large-scale machine learning on heterogeneous systems. arXiv.

[32] Cremer D., Pople J.A. (1975). General definition of ring puckering coordinates. J. Am. Chem. Soc..

[33] Alonso J.L., Lozoya M.A., Peña I., López J.C., Cabezas C., Mata S., Blanco S. (2014). The conformational behaviour of free D-glucose-at last. Chem. Sci..

[34] Mayes H.B., Broadbelt L.J., Beckham G.T. (2014). How Sugars Pucker: Electronic Structure Calculations Map the Kinetic Landscape of Five Biologically Paramount Monosaccharides and Their Implications for Enzymatic Catalysis. J. Am. Chem. Soc..

[35] Barnett C.B., Naidoo K.J. (2010). Ring Puckering: A Metric for Evaluating the Accuracy of AM1, PM3, PM3CARB-1, and SCC-DFTB Carbohydrate QM/MM Simulations. J. Phys. Chem. B.

[36] Homeyer N., Horn A.H.C., Lanig H., Sticht H. (2006). AMBER force-field parameters for phosphorylated amino acids in different protonation states: Phosphoserine, phosphothreonine, phosphotyrosine, and phosphohistidine. J. Mol. Model..

