# Supplementary material for: Improvement of the Force Field for β-d-Glucose with Machine Learning
Source: Molecules. 2021 Nov 5;26(21):6691. doi: 10.3390/molecules26216691 (PMC8588059; doi:10.3390/molecules26216691)
Supplement: Supplementary file 1 [file molecules-26-06691-s001.zip › molecules-1430650-supplementary.pdf]

# Supplementary Materials: Improvement of the Force Field for *β*-D-Glucose with Machine Learning

**Makoto Ikejo [1,*], Hirofumi Watanabe [2], Kohei Shimamura [1,3] and Shigenori Tanaka [1,*]**

## SM-1. Torsion parameters

The torsion parameters optimized as a result of this study are listed in the Table A.1 below. The "Torsion" row shows the bond expressed in terms of the atom type defined for each atom. In our force field, all carbon atoms belong to "cG" atom type, the oxygen atoms of the hydroxyl group belong to "oH" atom type, and the oxygen atoms in the six-membered ring belong to "oS" atom type. The hydrogen atoms of the hydroxyl group belong to "hO" atom type, the hydrogen atoms bonded to aliphatic hydrocarbon with 1 electron-withdrawing group ($H_2, H_3, H_4, H_5, H_{61}$, and $H_{62}$ in Fig. 1 of the main text) belong to "h1" atom type, and the hydrogen atom bonded to aliphatic hydrocarbon with 2 electron-withdrawing group ($H_1$ in Fig. 1 of the main text) belongs to "h2" atom type.

**Table S1.** Torsion parameters in Eq. 4 of the main text. The columns headed $V_1$ to $V_6$ have units of kcal/mol, and the columns headed $\gamma_1$ to $\gamma_6$ have units of degrees (°)

| Torsion | $V_1$ | $V_2$ | $V_3$ | $V_4$ | $V_5$ | $V_6$ | $\gamma_1$ | $\gamma_2$ | $\gamma_3$ | $\gamma_4$ | $\gamma_5$ | $\gamma_6$ |
|---|---|---|---|---|---|---|---|---|---|---|---|---|
| h1cGoHhO | 0.0468 | -0.0322 | 0.1851 | -0.0761 | -0.0500 | -0.0424 | 20.41 | 6.50 | -32.20 | 23.54 | 126.89 | -58.84 |
| cGoScGh1 | 0.0058 | -0.0062 | 0.2718 | -0.0045 | 0.0250 | -0.0347 | -2.76 | 14.77 | -18.64 | -5.11 | -13.81 | 22.97 |
| cGcGcGcG | 0.4376 | -0.0224 | -0.0135 | -0.0414 | 0.0125 | -0.0239 | -37.38 | -7.30 | 18.73 | 8.55 | -5.18 | 8.42 |
| cGcGcGh1 | 0.0341 | 0.0309 | 0.1496 | 0.0450 | -0.0371 | -0.1004 | 12.07 | -46.62 | 47.53 | -0.12 | -29.30 | 89.36 |
| hOoHcGcG | -0.0814 | 0.0716 | 0.2341 | 0.0585 | -0.0095 | -0.0844 | 40.22 | 96.96 | 6.57 | 45.02 | 30.89 | -15.98 |
| h2cGoScG | 0.0028 | 0.5981 | 0.0856 | 0.0390 | 0.0545 | -0.0334 | 1.76 | 1.07 | -29.44 | -51.88 | 42.77 | -19.74 |
| h2cGcGcG | 0.0022 | -0.0151 | 0.1703 | 0.0076 | 0.0001 | -0.0310 | -1.72 | -2.88 | 25.91 | -19.11 | 20.06 | 15.44 |
| oHcGcGh1 | -0.0049 | -0.0275 | 0.0203 | -0.0502 | -0.0462 | -0.0657 | -6.04 | 32.15 | -16.16 | 27.44 | -32.52 | -39.24 |
| oScGcGh1 | 0.0163 | 0.0210 | 0.0662 | -0.0654 | -0.0653 | -0.0458 | 23.12 | -20.06 | 28.21 | 10.57 | -1.68 | 71.37 |
| oScGcGoH | -1.1223 | 0.2221 | 0.0255 | 0.0344 | 0.0721 | -0.0400 | -17.52 | -44.13 | 24.69 | -3.36 | -10.78 | 11.54 |
| oHcGcGcG | -0.0128 | 0.0240 | 0.0891 | -0.0573 | 0.0455 | -0.0805 | 11.62 | 36.90 | -43.50 | -101.04 | -40.03 | 5.16 |
| h1cGcGh1 | -0.0139 | 0.0136 | 0.1425 | 0.0180 | 0.0314 | 0.0043 | -8.69 | 0.88 | 7.55 | 6.03 | -27.64 | 35.25 |
| hOoHcGoS | 0.0436 | 0.0797 | 0.2186 | -0.0442 | -0.0837 | -0.0691 | 6.57 | 5.96 | -8.58 | 3.46 | -34.15 | -82.49 |
| oHcGcGoH | -0.0977 | 0.9428 | 0.5479 | 0.0199 | 0.0073 | -0.0499 | 11.57 | -29.31 | -24.23 | -13.69 | -1.42 | -14.79 |
| h2cGcGoH | 0.0068 | 0.0220 | 0.0769 | 0.0101 | -0.0177 | -0.0324 | -6.70 | 10.01 | 29.27 | 23.47 | -7.71 | 26.67 |
| cGcGoScG | -0.0016 | 0.0053 | 0.1452 | -0.0458 | -0.0902 | -0.0829 | 9.46 | -19.17 | 13.16 | 9.83 | -14.48 | 28.28 |
| oScGcGcG | -0.2596 | 0.0189 | 0.0217 | 0.0141 | -0.0207 | -0.0293 | -43.64 | 7.55 | 21.81 | 33.82 | -17.65 | 6.57 |
| oHcGoScG | 1.0768 | 1.3802 | 0.9481 | 0.0248 | 0.0464 | -0.0348 | -14.26 | 18.27 | -30.16 | 7.01 | -63.15 | -12.03 |
| hOoHcGh2 | -0.0411 | -0.0818 | 0.2318 | 0.0306 | 0.0802 | -0.0742 | -50.79 | 60.36 | -11.21 | -45.01 | 25.79 | -74.73 |
| h2cGcGh1 | -0.0056 | -0.0033 | 0.1862 | -0.0068 | 0.0129 | -0.0257 | 7.18 | 12.49 | 26.36 | 6.13 | 21.18 | 18.18 |

## SM-2. Results of structual sampling

The results of structural sampling with GLYCAM06 force field are shown below. Figure S1 shows the correspondence between the conformers of the six-membered ring structure of $\beta$-glucose and the Cremer-Pople coordinates. The $^4C_1$ structure, which is generally considered to be the most stable, exists around $\theta = 0°$, while some metastable structures correspond to the $\theta = 90°$ and $\theta = 180°$ regions. The sampling results at 300 K are shown in Fig. S2. At 300K, almost all the structures were sampled with only $^4C_1$ structures. On the other hand, the sampling results at 600 K are shown in Fig. S3. At 600 K, a wider range of conformers were obtained. In Figs. S2 and S3, all 20,000 structures obtained from MD simulations at 300 and 600 K were plotted, respectively, while in the structural optimization by QM (Sec. 2.2) 1,000 structures obtained by increments of 20 were used as initial structures.

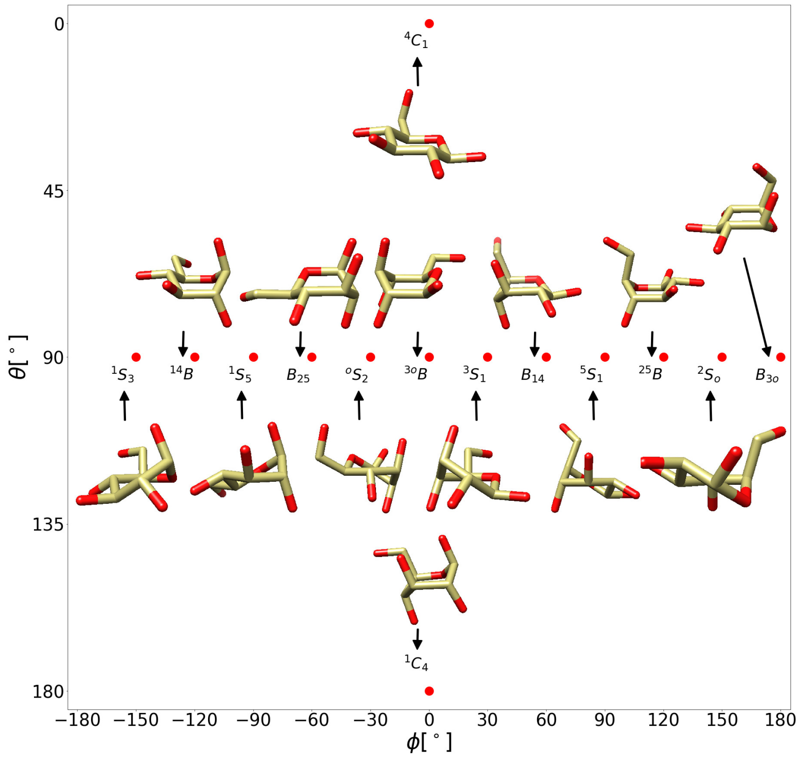


**Figure S1.** Mercator projections of the CP puckering coordinates of $\beta$-glucose conformers. The red dots represent the typical conformers.

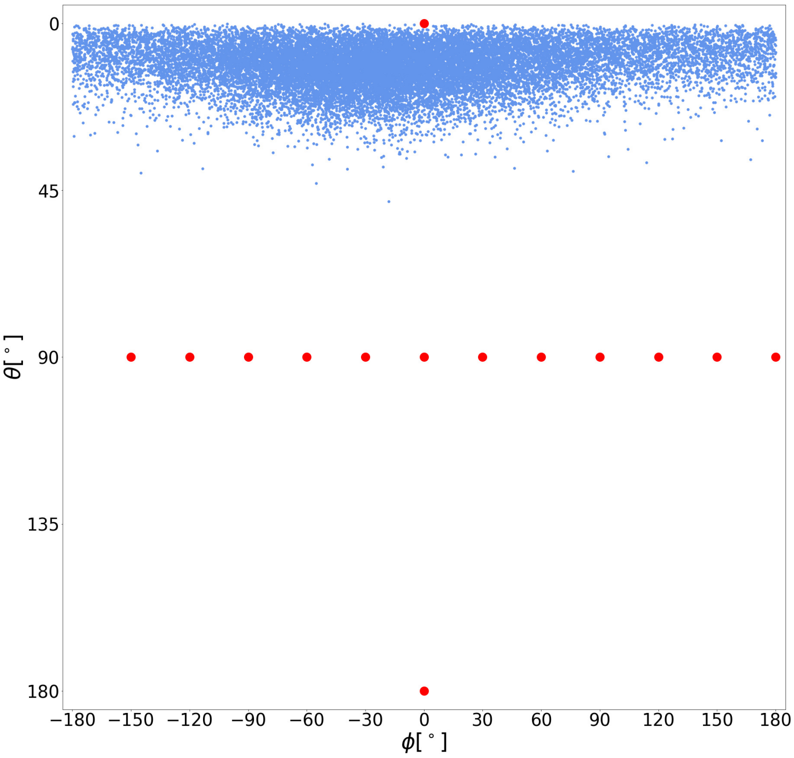


**Figure S2.** Mercator projections of the CP puckering coordinates of structural sampling at 300K. The blue dots represent the structures obtained by MD, and the red dots represent the conformers shown in Fig. S1

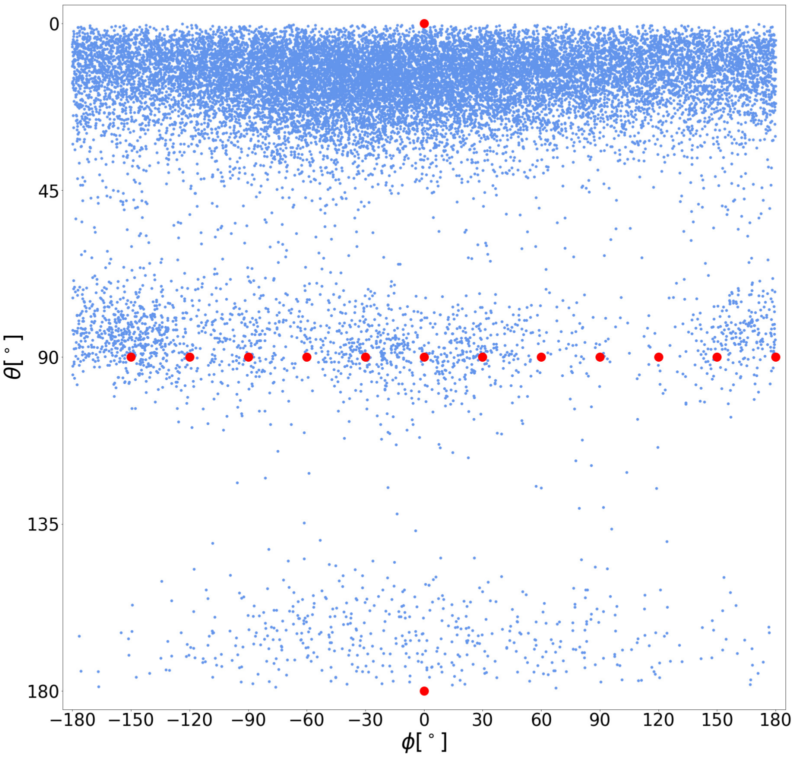


**Figure S3.** Mercator projections of the CP puckering coordinates of structural sampling at 600K. The blue dots represent the structures obtained by MD, and the red dots represent the conformers shown in Fig. S1

## SM-3. RESP Charge distribution

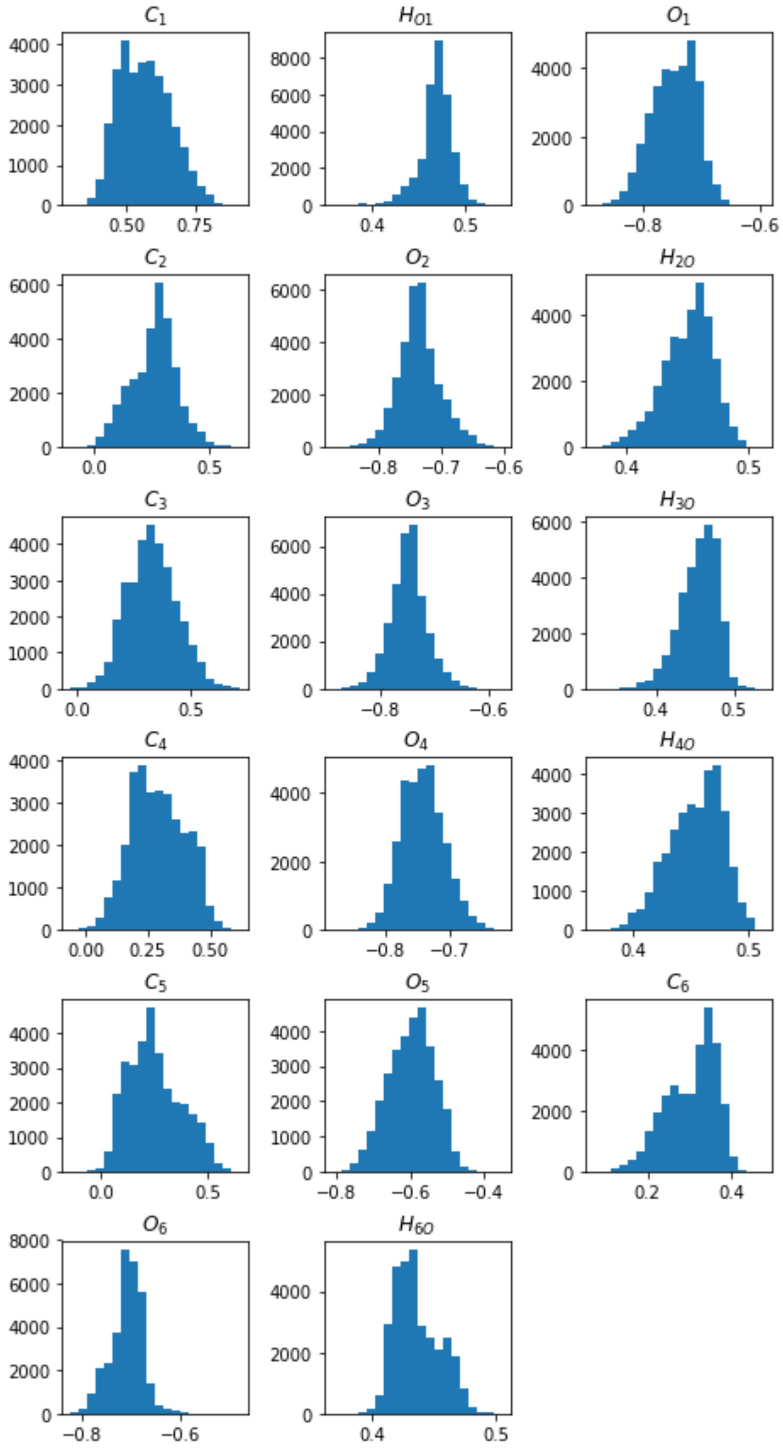


**Figure S4.** Distribution of RESP charges for all 31,899 structures. Atomic labels correspond to Fig. 1 in the main text.

## SM-4. Examination on overfitting

We verified that no overfitting occurred during the parametric optimization. Evaluations of $C$ and $\langle \mathrm{REE}^2 \rangle$ in Eq. 7 for training and test sets were shown in Fig. S5, which confirmed that no overfitting occurred during the parametric optimization. Furthermore, the REE distributions for the training and test sets and the scatter plot of $\Delta E_{\mathrm{QM}} = E_{\mathrm{QM},i} - E_{\mathrm{QM,ref}}$ versus $\Delta E_{\mathrm{MM}} = E_{\mathrm{MM},i} - E_{\mathrm{MM,ref}}$ are shown in Fig. S6.

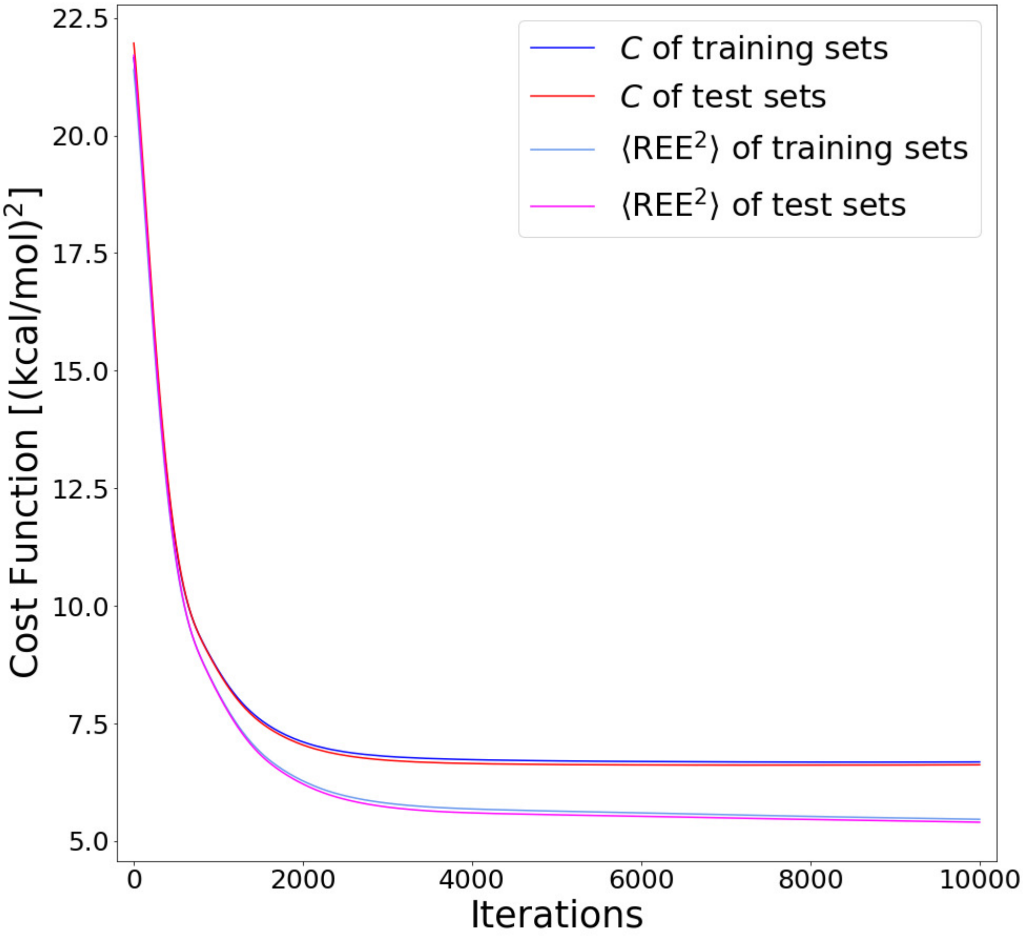


**Figure S5.** Evaluation of cost function. The abscissa refers to fitting iterations, and the ordinate is $C$ and $\langle \mathrm{REE}^2 \rangle$, where blue and red colors show $C$ of Eq. 7 for training and test sets, respectively, and light blue and pink colors show $\langle \mathrm{REE}^2 \rangle$ of Eq. 7 for training and test sets, respectively.

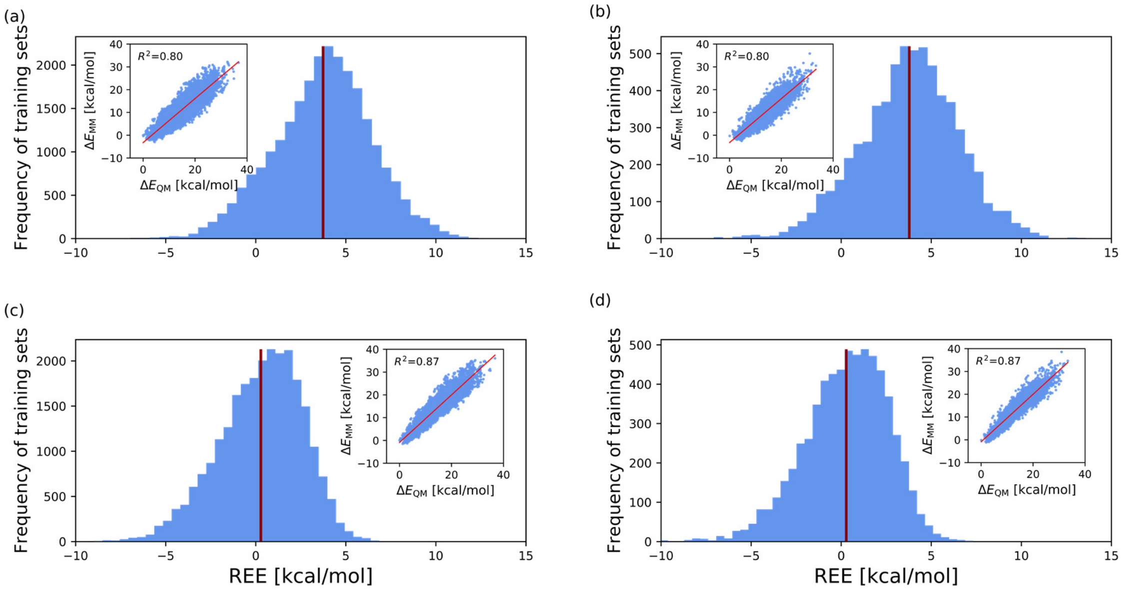


**Figure S6.** Histogram of REEs for training and test set structures, with REE on the abscissa and the number of samples on the ordinate. The red bars indicate the average values of REE. The scatter diagram in the inset is also plotted with $\Delta E_{QM}$ on the abscissa and $\Delta E_{MM}$ on the ordinate. (a) and (b) show the results obtained using the GLYCAM06 force field for computing the MM energies with training and test set structures, respectively. (c) and (d) show the results obtained using the the presently proposed force field for computing the MM energies with training and test set structures, respectively.

**SM-5. Definition of conformers of $^4C_1$ structure**

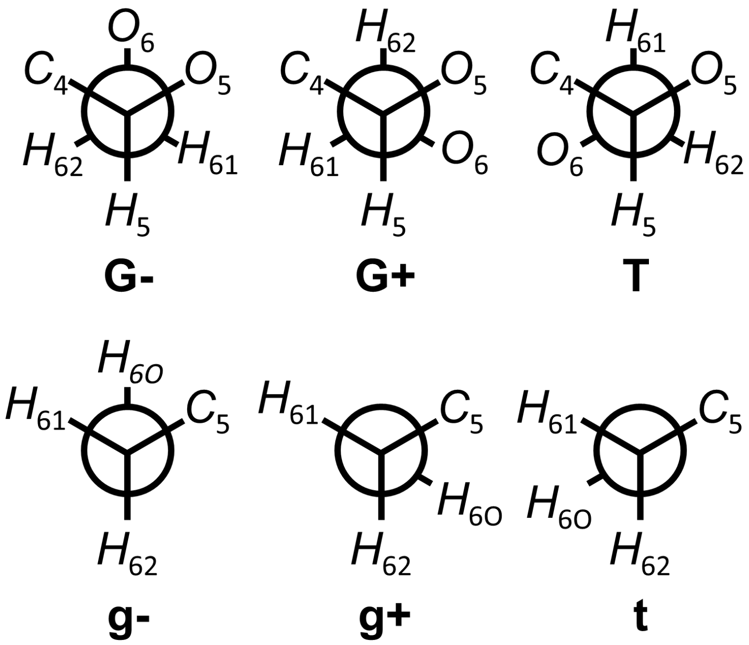


**Figure S7.** Newman projections of the conformations of the hydroxymethyl group around $C_5$-$C_6$ and $C_6$-$O_6$ bonds.

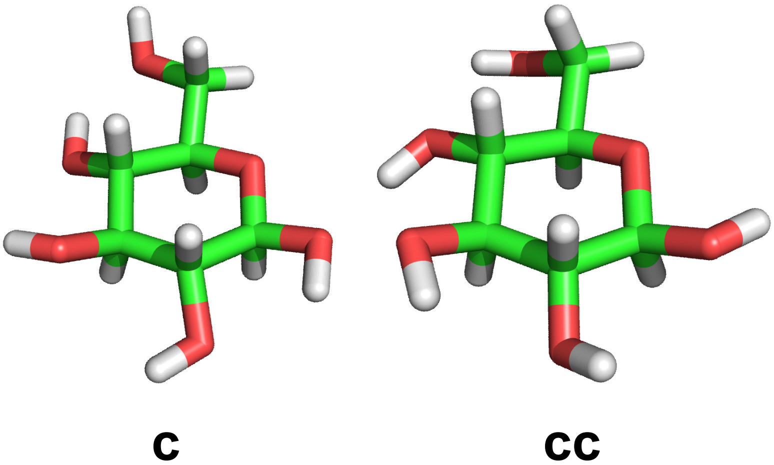


**Figure S8.** The clockwise (C) and counter-clockwise (CC) conformers of $\beta$-glucose.

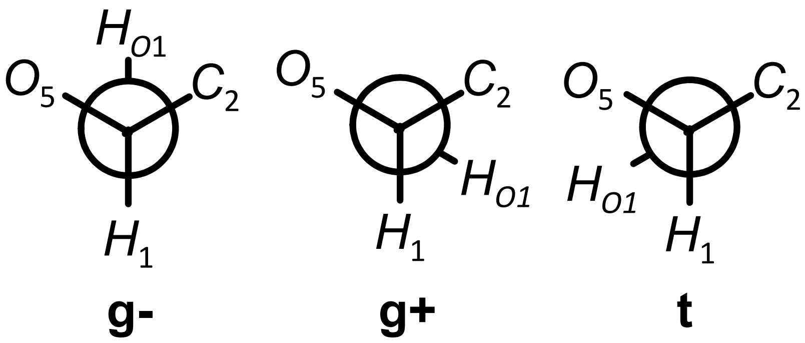


**Figure S9.** Newman projections of the conformations of the hydroxyl group around $C_1$-$O_1$ bonds.

**SM-6. Parametric optimization code**

The code used for parametric optimization in this work has been uploaded to the GitHub server: https://github.com/mmikejo/optimization_force_field_parameters_for_carbohydrate The comment out in the code is currently written in Japanese, but will be changed to English in the future. On the other hand, since the various input data used for the calculations are too large to be uploaded on GitHub, they have been uploaded to the Google Drive: https://drive.google.com/drive/folders/1jAbYIfcm9tVxtbwxN_IMbM1gdB1LOR6T?usp=sharing
